# Venous thromboembolism in head and neck cancer surgery

**DOI:** 10.1186/s41199-016-0014-9

**Published:** 2016-11-01

**Authors:** Faisal I. Ahmad, Daniel R. Clayburgh

**Affiliations:** Department of Otolaryngology- Head & Neck Surgery, Oregon Health and Science University, 3181 SW Sam Jackson Park Road, PV01, Portland, OR 97239 USA

**Keywords:** Venous Thromboembolism, Fondaparinux, Neck Cancer Patient, Abnormal Pulmonary Function, Microvascular Reconstruction

## Abstract

**Background:**

Venous thromboembolism (VTE) is a major cause of perioperative morbidity and mortality. Historically, otolaryngology surgery has been seen as very low risk of VTE, given the relatively short procedures and healthy patient population. However, head and neck surgery patients have multiple additional risk factors for VTE compared to general otolaryngology patients, and only recently has research been directed at examining this population of patients regarding VTE risk.

**Review:**

VTE has long been recognized as a major issue in other surgical specialties, with VTE rates of 15–60 % in some specialties in the absence of prophylaxis with either mechanical compression or anticoagulation. Multiple large-scale retrospective studies have shown that the incidence of VTE in otolaryngology patients is quite low, ranging between 0.1 and 1.6 %. However, these studies indicated that head and neck cancer patients may have an increased risk of VTE. Further retrospective studies focusing on head and neck cancer patients found a VTE rate of approximately 2 %, but one study also found a suspected VTE rate of 5.6 % based on clinical symptoms, indicating that retrospective studies may underreport the true incidence. A single prospective study found a 13 % risk of VTE after major head and neck surgery. Furthermore, risk stratification using the Caprini risk assessment model demonstrates that the highest risk patients may have a VTE risk of 18.3 %, although this may be lowered (but not eliminated) through the use of appropriate prophylactic anticoagulation.

**Conclusion:**

VTE is likely a more significant concern in head and neck surgery patients than previously realized. Appropriate prophylaxis with mechanical compression and anticoagulation is essential; risk stratification may serve as a useful tool to identify head and neck cancer patients at highest risk for VTE.

## Background

Venous thromboembolism (VTE), which includes deep vein thrombosis (DVT) and pulmonary embolism (PE), is a common problem in hospitalized patients and can cause significant morbidity and mortality. This condition is responsible for 5 to 10 % of all hospital deaths, and is estimated to affect as many as 600,000 patients a year in the United States [1]. Surgery increases the risk of VTE nearly 20-fold [2]. In the absence of prophylaxis, the estimated incidence of VTE among general surgery patients is 15 to 40 % and is notably higher at 40 to 60 % in orthopedic surgery patients [3]. In the setting of cancer, surgery further doubles this risk compared to patients without cancer [4]. The incidence of VTE also varies depending on the type of cancer, with malignancies of the bone, ovary, brain and liver/pancreas associated with the highest incidences [5, 6].

In a 2003 study including over 7 million patients in 944 hospitals in the United States [7], VTE was the second most common serious post-operative complication. On average, a post-operative episode of VTE increases the patient length of stay by over 5 days, resulting in excess charges of $21,000, and has a 6.56 % excess mortality rate. Furthermore, outpatient anticoagulation after VTE is expensive, with 1 year of therapeutic anticoagulation and monitoring costing approximately $33,000 [8]. Nevertheless, post-operative VTEs are highly preventable and represent the most common cause of preventable 30-day surgical mortality in patients undergoing cancer resection [6, 9]. Consequently, chemoprophylaxis with anticoagulants such as low-molecular-weight heparin or fondaparinux is frequently recommended in post-operative patients [3].

The risk of VTE in general otolaryngology has been considered to be very low, as procedures are often done on an outpatient basis and there is no associated immobilization or impairment of ambulation. Prior retrospective studies of general otolaryngology patients have demonstrated a low risk of VTE, between 0.1 and 2.4 % [10–13]. The bleeding risk associated with VTE chemoprophylaxis also presents a unique set of complications in head and neck surgery, including airway compromise, wound complications, and failure of microvascular reconstruction. As such, compliance with VTE chemoprophylaxis guidelines has been low among head and neck surgeons [14]. However, patients with head and neck cancer are intrinsically different than otherwise healthy general otolaryngology patients, and often have multiple risk factors for VTE development, including malignancy, pulmonary comorbidity, large, complex surgeries, and other medical problems. More recent studies have indicated that head and neck surgery patients demonstrate substantially higher rates of VTE, reaching nearly 20 % in the highest-risk subgroups [12]. The purpose of this review is to examine the literature on incidence and prophylaxis of VTE in head and neck surgery patients.

### Venous thromboembolism pathophysiology and prophylaxis

In the 1800s, multiple pathologic factors—abnormal blood flow or stasis, endothelial injury, and hypercoagulability—were described as the etiologic agents for venous thrombosis. Dubbed Virchow’s triad, this provides a framework for understanding thrombus formation. Although an extensive list of risk factors are known for VTE (Table 1), all these risk factors can be distilled down to affecting one or more of these central principles of Virchow’s Triad. A review of the molecule underpinnings of coagulation are outside the scope of this paper, but have been reviewed extensively in other publications [15–19].

**Table 1:** Risk factors for venous thromboembolism

Surgery	
Trauma	
Immobility/paresis	
Cancer	
Cancer therapy	
Venous compression	
Previous VTE	
Increasing age	
Pregnancy/postpartum	
Oral contraceptive/HRT	
Estrogen receptor modulators	
Erythropoiesis-stimulating agents	
Acute medical illness	
Inflammatory bowel disease	
Nephrotic syndrome	
Myeloproliferative disorders	
Paroxysmal nocturnal hemoglobinuria	
Obesity	
Central venous catheterization	
Inherited/acquired thrombophilia	

Nevertheless, while the inciting mechanisms in situations of vascular injury are relatively well known, it is somewhat less clear how thrombus formation may occur in the setting of an intact endothelium, as occurs with venous thromboembolism. In this setting, thrombus formation is likely much more dependent upon inflammation, stasis, and/or hypercoagulability. Both cancer and surgery are well-known to induce pro-inflammatory states and thus induce hypercoagulability, putting post-surgical patients, and oncologic surgical patients in particular, at much higher risk for VTE development. Given the significant morbidity and mortality that may be caused by VTE, prophylaxis against VTE has been widely studied. Methods of VTE prophylaxis can generally be divided into two broad categories: mechanical and pharmacologic. Pharmacologic prophylaxis includes anticoagulants such as unfractionated heparin, low-molecular weight heparin, fondaparinux, and warfarin. While these drugs increase bleeding risk after surgery, they to do provide significant protection from the development of VTE. There is no evidence to support the use of inferior vena cava filters in VTE prophylaxis; in fact, not only do these filters increase the total procedure cost, they may actually raise the risk of DVT [20]. The American College of Chest Physicians was created guidelines for the use of thromboprophylaxis, and this article provides a thorough review of VTE prophylaxis modalities and their relative risks and benefits [9].

### VTE in cancer patients

The association between thromboembolism and malignancy has been recognized for over 150 years. In 1865 Armand Trousseau described cancer-associated thrombosis and a unique alteration of the blood, thereby recognizing the hypercoagulable state induced by malignancy [21]. Approximately 20 % of cancer patients experience thrombosis at some point, and thrombosis may be found on autopsy in up to 60 % of patients that die of cancer [22, 23]. In a retrospective study from the Netherlands, Blom et al. [5] found that the risk of VTE was 12.3 per 1000 in cancer patients compared to 2 per 1000 for the general population. Specifically, the cumulative incidence of VTE was highest for cancers of bone, ovary, brain and pancreas. Interestingly, they also found patients with distant metastases had a nearly two-fold increase in relative risk VTE compared to those without metastatic disease. Unfortunately, data for head and neck cancer was not available in sub-group analysis, likely due to the rarity of head and neck cancer compared to more common types.

The six-fold increased incidence of venous thrombosis in cancer patients can be attributed to baseline patient characteristics, tumor factors, and factors relating to oncologic treatment. Risk factors and comorbid conditions that are associated with head and neck cancer also play a role in VTE formation and include older age, tobacco use, obesity, and abnormal pulmonary function (e.g. COPD) [3, 24]. Although the exact mechanisms by which malignancy increases the incidence of VTE formation have not been completely elucidated, it is believed that inflammatory cytokines may induce endothelial injury and promote a hypercoagulable state. These procoagulants may be directly released by the tumor, or through induction of procoagulant production by native cells.

### Venous thromboembolism in other surgical specialties

Data regarding the incidence of VTE in other surgical specialties and evidence for chemoprophylaxis is abundant, and an exhaustive review of this literature is beyond the scope of this paper. Historically, prospective screening studies have demonstrated that the incidence of asymptomatic VTE is 15–40 % in abdominal surgery and 40–60 % in orthopedic surgery in the absence of VTE prophylaxis [3]. However, multiple recent studies have demonstrated an overall incidence of 1–2 % with modern prophylaxis regimens among a heterogeneous mix of surgical procedures [25–27]. A recent retrospective study using the American College of Surgeons National Surgical Quality Improvement Program examined VTE after cancer surgery [6]. This study found a 1.6 % incidence of VTE in over 44,000 patients with 33.4 % of these occurring after discharge. Furthermore, patients who experienced a VTE had a statistically significant 6-fold (8.0 vs 1.3 %) increase in mortality. Though this study could not attribute causality between VTE and mortality, other studies have demonstrated that VTE is the most-common cause of 30-day postoperative mortality in cancer surgery patients [27]. In a systematic review of 25 randomized trials comparing combined chemoprophylaxis and compression devices with compression alone, Zereba et al. [28] demonstrated a 44 % reduction in the risk of DVT with combined therapy. However, the use of chemoprophylaxis also increased the relative risk of bleeding by 74 %. Thus, current clinical practice guidelines recommend the use of anticoagulation (either heparin or low molecular weight heparin) along with compression devices in all patients with malignancy undergoing major surgery, unless a high risk of bleeding exists [29].

Although prophylaxis is often considered for patients during the acute inpatient hospitalization, there is data to suggest that anticoagulation after discharge may also be beneficial. A prospective, placebo-controlled, double-blind trial in which patients undergoing abdominal surgery for cancer were randomized to therapy with enoxaparin for 4 weeks versus only 1 week showed a 60 % decrease in venographically demonstrated thrombosis with longer therapy [30]. Furthermore, this risk reduction in thromboembolic events was durable even 3 months after surgery. Accordingly, the American Society of Clinical Oncology in their 2014 Clinical Practice Guideline recommends chemoprophylaxis in patients with active cancer undergoing major surgery starting pre-operatively and continuing at least 7–10 days with consideration of extending therapy for up to 4 weeks [29].

### Venous thromboembolism in head and neck cancer surgery

Although it is well known that the incidence of VTE for cancer patients is increased compared patients without cancer, data for the incidence of VTE in head and neck cancer patients and the need for chemoprophylaxis is extremely limited. As previously mentioned, early assumptions regarding VTE in surgical head and neck cancer patients was extrapolated from general otolaryngology patients. In a study from 1998, Moreano et al. [31] examined almost 13,000 patients at a tertiary care center and found a VTE rate of 0.3 % in general otolaryngology patients and 0.6 % in head and neck surgery patients. In another more recent retrospective study [11], approximately 6000 otolaryngology patients at a tertiary care center were examined and only six cases (0.1 %) of symptomatic VTE were discovered. However, all observed VTE occurred in head and neck surgery patients; 824 total patients had surgery for malignancy and the 6 observed VTE yielded a rate of 0.6 %.

Although these studies hinted that head and neck surgery patients may be at higher risk for VTE than general otolaryngology patients, many previously felt that head and neck surgery patients may be at substantially lower risk for VTE than standard surgical oncology patients. Unlike surgery of the chest, abdomen, pelvis, or lower extremities that may severely limit patient mobility for an extended period of time, head and neck surgery patients are often able to get out of bed and ambulate quite soon after surgery. Furthermore, the risks of anticoagulation in these patients may be higher, as bleeding complications in the head and neck may have more profound consequences. Hemorrhage into the airway can rapidly prove life-threatening, and a hematoma in the confines of the neck may easily compromise vascular anastomoses for microvascular reconstructions. Thus, there is concern that the risk/benefit ratio for postoperative anticoagulation may not be as favorable in head and neck surgery patients as that seen in other surgical fields. Accordingly, the use of VTE prophylaxis by head and neck surgeons has been exceedingly variable. In a survey of over 600 practicing otolaryngologists, significant variability was seen in the use of VTE prophylaxis, with 74 % of respondents routinely prescribing postop SCD use, 38 % using postop compression stockings, 36 % using low molecular weight heparin, and 16 % using heparin [32].

Despite these concerns regarding VTE prophylaxis in head and neck cancer patients, in many areas guidelines developed in other specialties regarding the need for VTE prophylaxis have been applied to head and neck surgery patients. However, there is very little high-quality data that specifically addresses VTE in head and neck surgery patients; most studies are retrospective in nature. A retrospective study from Australia [33] examined the incidence of VTE in 1018 patients undergoing oncologic head and neck surgery. In this cohort, 56 % of patients received VTE chemoprophylaxis, while the remainder did not. Although the rate of VTE was 0 % in both groups, the group receiving chemoprophylaxis had a six-fold increase in bleeding and hematoma rate. Garritano et al. [13] assessed 268 patients undergoing inpatient procedures for head and neck cancer and found an incidence 1.1 % (3/268). A study from Pakistan [34] examined rates of VTE in 413 patients undergoing surgery for head and neck cancer. Their overall rate of VTE was 2.9 % despite routine prophylaxis with low molecular weight heparin. They also found that patients who developed VTE typically had longer cases (10.8 versus 6.9 h), and involved reconstruction with a pedicled or free flap.

However, there is an inherent issue with these retrospective studies, in that they may underreport the true incidence of venous thromboembolism. These studies would necessarily only detect those VTE that became clinically evident around the time of surgery, and may miss patients with either clinically silent VTE, unrecognized/misdiagnosed VTE, or those that did not manifest clinical symptoms until after hospital discharge. The possibility of significant underreporting of VTE in retrospective studies was raised by the study by Thai et al. [35], which retrospectively examined 134 head and neck cancer patients undergoing resection and microvascular reconstruction. They found a 1.4 % (2/134) rate of confirmed VTE as documented in the chart. However, when other clinical symptoms were assessed that could be the result of VTE (i.e. leg swelling, sudden death, or other possible sequelae of VTE without evidence of VTE assessment in the medical record), the rate of possible VTE rose to 5.8 % (8/134). Additionally, they showed that strong predictors of a patient developing a VTE included prior VTE, blood transfusion, high body mass index, and older age. This study highlights the difficulty with retrospective data for VTE assessment, as it suggests that in certain high-risk groups (i.e. major ablative surgery with microvascular reconstruction) the rate of VTE may be higher than initially assumed.

The clear next step required to better understand VTE risk in head and neck surgery is high-quality prospective studies. To date, only a single prospective study has been conducted to assess VTE incidence in head and neck cancer surgery [24]. This study enrolled 100 consecutive patients undergoing major surgery to treat head and neck cancer (defined as anticipated postoperative length of hospital stay >4 days, typically involving microvascular reconstruction or other large procedures such as total laryngectomy). Patients received routine clinical examination for VTE, as well as lower extremity Doppler ultrasound evaluation on postoperative day 2 or 3. Patients were also followed clinically for 30 days after surgery. This demonstrated a 13 % overall rate of VTE in this patient population – much higher than that seen in previous retrospective studies. More specifically, eight patients had clinically significant VTE (7 DVT and 1 PE) and 5 patients had asymptomatic lower extremity superficial VTE. Although routine chemoprophylaxis was not part of the study protocol, 14 patients received anticoagulation for other indications and had a higher rate of bleeding complications (30.1 versus 5.6 %) compared to those without anticoagulation. While this study was designed as a pilot study rather than a full assessment of VTE risk, and is not adequately powered to provide further detail on VTE risk based on tumor type, surgical procedure, or other factors, it does suggest that the true incidence of VTE in high-risk head and neck surgery patients is higher than previously reported and may be more similar to other high-risk surgery groups. Further work will be needed to define the risks and benefits of routine chemoprophylaxis on the incidence of VTE in surgical head and neck cancer patients, and provide further granularity regarding VTE risk in the head and neck surgery population, such as differences between tumor histology, subsites, procedures, and other factors.

### Risk stratification in head and neck cancer patients

Given the potential risks of VTE chemoprophylaxis in head and neck surgery patients, alongside the wide variation in reported VTE incidence rates, the optimal strategy for VTE prevention in head and neck patients may be to specifically target chemoprophylaxis towards the patients at highest risk of VTE, and spare patients at low risk from the potential bleeding complications associated with anticoagulation. Thus, there is significant interest in risk stratifying patients for VTE. In 2001 Caprini et al. [36] proposed a risk assessment model (RAM) for stratifying the risk of VTE in both surgical and non-surgical patients. The Caprini RAM predicts the risk of VTE by adding together points for various VTE risk factors (Table 2). In this model, the points for each risk factor are weighted based on their association with developing VTE. The Caprini RAM has been validated in a retrospective cohort of approximately 8000 general, vascular, and urologic surgery inpatients [37]. The risk of developing a VTE was strongly associated with the Caprini score and is demonstrated in Table 3. Risk factors that are particularly germane to surgical head and neck cancer patients include obesity (BMI > 25), serious lung disease or abnormal pulmonary function, advancing age, malignancy and major surgery (>45 min). It is important to note that with this tool, the difference between major and minor surgery is defined only by time of greater than or less than 45 min, rather than by specific procedure. Based on this definition, nearly all ablative head and neck procedures would meet the definition of major surgery.

**Table 2:** Caprini Risk Assessment Model

Each Risk Factor Represents 1 Point	Each Risk Factor Represents 2 Points
• Age 41–60 years • Swollen legs • Varicose veins • Obesity (BMI > 25) • Minor surgery planned • Sepsis (<1 month) • Serious lung disease including pneumonia (<1 month) • Acute myocardial infarction • Congestive heart failure (<1 month) • Medical patient currently at bed rest • History of inflammatory bowel disease • History of major surgery (<1 month) • Abnormal pulmonary function (COPD) • Pregnancy or postpartum (<1 month) • Oral contraceptive or hormone replacement therapy • History of unexplained stillborn infant or recurrent spontaneous abortion (>3), premature birth with toxemia or growth restricted infant	• Age 61–74 years • Arthroscopic surgery • Malignancy (present or previous) • Laparoscopic surgery (>45 min) • Patient confined to bed (>72 h) • Immobilizing plaster cast (<1 month) • Central venous access • Major surgery (>45 min) Each Risk Factor Represents 3 Points • Age 75 years or older • History of DVT/PE • Positive Factor V Leiden • Elevated serum homocysteine • Heparin-induced thrombocytopenia • Elevated anticardiolipin antibodies • Other congenital or acquired thrombophilia • Family history of thrombosis • Positive prothrombin 20210A • Positive Lupus anticoagulant Each Risk Factor Represents 5 Points • Stroke (<1 month) • Multiple trauma (<1 month) • Elective major lower extremity arthroplasty • Hip, pelvis or leg fracture (<1 month) • Acute spinal cord injury (<1 month)
Total:

**Table 3:** Incidence rate of VTE without routine anticoagulation based on cumulative Caprini score [37]

Cumulative Caprini score	Incidence rate of VTE
0–1	0.00 %
2	0.70 %
3–4	0.97 %
5–6	1.33 %
7–8	2.58 %
9+	6.51 %

While the Caprini RAM was not developed specifically for surgery, it has actually been studied most extensively as a risk stratification tool in head and neck surgery. Shuman et al. [12] retrospectively assigned Caprini scores to 2016 otolaryngology inpatients and then examined their 30-day rate of VTE. Notably, none of these patients received thromboprophylaxis with heparin or low molecular weight heparin. In this study, 88 % of patients were assigned as having high (total score 3–4) or highest (total score >5) risk, which likely reflects in inpatient status of this population. Although the overall 30-day rate of VTE was only 1.3 %, patients with a Caprini score of <5 had a 0.5 % incidence of VTE compared to 2.4 % for patients with a score of 7–8 and 18.3 % for patients with a score >8. Evaluation of specific risk factors revealed that patients with higher scores were those with cancer, chronic obstructive pulmonary disease, recent stroke, central access, and infections. The same group later compared the above results to a cohort of 1482 otolaryngology patients that had received chemoprophylaxis for VTE [38]. In the cohort that received chemoprophylaxis, the rate of overall VTE was similar at 1.2 %. However, when patients were stratified by Caprini score, a non-statistically significant reduction in VTE rate was seen with chemoprophylaxis in patients with scores between 7–8 (1.9 versus 2.4 %), and >8 (10.7 versus 18.3 %).

In another study, Yarlagadda et al. [39] retrospectively reviewed 704 otolaryngology patients that received appropriate thromboprophylaxis (early ambulation, compression devices, chemoprophylaxis, or a combination) based on institutional standards according to their Caprini scores. In this study, the average Caprini score was 5.7 and the overall rate of VTE was 2.1 %. As expected, patients with higher Caprini scores were found to have higher rates VTE. The rates of VTE for patients with Caprini scores less than 6, between 7 and 8, and greater than 9 were 0.0, 3.0, and 13.1 % respectively. This study also found that patients with a history of prior VTE, malignancy, bedbound beyond 72 h, and congestive heart failure had an association with increased risk of VTE.

These studies suggest that the Caprini score may be a useful tool to risk stratify head and neck surgery patients. There is a positive correlation between Caprini score and incidence of VTE, and this tool may successfully identify those patients most likely to benefit from VTE chemoprophylaxis. Although these studies were primarily limited by the small numbers of patients available with high Caprini scores, they do suggest that directing chemoprophylaxis towards the highest risk patients may be a viable strategy to decrease the VTE rate, while chemoprophylaxis of patients with low Caprini scores is likely unnecessary. Equally important in this data is the realization that while appropriate chemoprophylaxis may reduce VTE risk, it cannot entirely eliminate it; there remains a subset of high-risk patients that will develop VTE despite appropriate prophylaxis. Thus, VTE in head and neck surgery cannot be considered a “never” event despite appropriate preventative measures; clinical vigilance for this complication and timely recognition and treatment will continue to be crucial for head and neck surgeons to prevent the sequelae of VTE.

## Conclusions

Venous thromboembolism is a major source of perioperative morbidity and mortality that is largely preventable. It accounts for approximately 10 % of hospital deaths annually [3], and patients that survive are at risk for further complications. Although both surgery and cancer are major risk factors for VTE, compliance with chemoprophylaxis guidelines has traditionally been low in head and neck surgery. This is largely driven by the historically perceived low risk of VTE in head and neck patients and potential for significant complications with anticoagulation. Recent studies have demonstrated that even though most otolaryngology patients are considered to be at low risk for VTE, head and neck cancer patients constitute a unique group with different VTE risks (summarized in Table 4). Specifically, the rate of VTE in head and neck cancer patients is much higher than has been reported in previous retrospective studies, particularly in the highest-risk patients. Identification of patients at the highest risk of VTE is vital to appropriately direct surveillance and prevention resources. As such, risk assessment models like the Caprini RAM may be useful to identify those most likely to benefit from chemoprophylaxis. However, while chemoprophylaxis may reduce the risk of VTE in high-risk groups, the risk is not eliminated, and there is an associated increase in bleeding risk. Further large-scale prospective trials will be necessary to make definitive recommendations on risk stratification and VTE prophylaxis in head and neck cancer patients.

**Table 4:** Studies of VTE Incidence in Otolaryngology

Source	Study design	Study population	Hospital status	Routine anticoagulation	VTE incidence, %
Jain et al. [40] (*n* = 6788)	Retrospective	General otolaryngology	Inpatient and outpatient	No	0.1
Chen et al. [41] (*n* = 48,028)	Retrospective	General otolaryngology	Inpatient and outpatient	No	0.1
Lin et al. [42] (*n* = 330,629)	Retrospective	General otolaryngology	Inpatient and outpatient	No	0.3
Moreano et al. [31] (*n* = 12,805)	Retrospective	General otolaryngology	Inpatient and outpatient	No	Overall, 0.5 Head and neck surgery, 1.0
Lee et al. [10] (*n* = 10,176)	Retrospective	General otolaryngology	Inpatient and outpatient	No	0.15
Innis et al. [11] (*n* = 6122)	Retrospective	General otolaryngology	Inpatient and outpatient	No	Overall, 0.1 Cancer patients, 0.6
Garritano et al. [13] (*n* = 5616)	Retrospective	General otolaryngology	Inpatient and outpatient	No	0.085
Shuman et al. [12] (*n* = 2016)	Retrospective	General otolaryngology	Inpatient	No	Overall, 1.6 Caprini score > 8, 18.3
Yarlagadda et al. [39] (*n* = 704)	Retrospective	General otolaryngology	Inpatient	Yes, depending on Caprini score	Overall, 2.1 Caprini score > 8, 13.1 %
Hennessey et al. [43] (*n* = 93,663)	Retrospective	Head and neck cancer	Inpatient	Unknown	2
Thai et al. [35] (*n* = 134)	Retrospective	Head and neck cancer	Inpatient	No	Confirmed VTE, 1.4 Suspected VTE, 5.8
Chen et al. [44] (*n* = 1591)	Retrospective	Head and neck cancer	Inpatient	Yes	0.70
Ali et al. [34] (*n* = 413)	Retrospective	Head and neck cancer	Inpatient	Yes	2.9
Clayburgh et al. [24] (*n* = 100)	Prospective	Head and neck cancer	Inpatient	No	Overall, 13 Clinically significant, 8

## Abbreviations

BMI: Body mass index
COPD: Chronic obstructive pulmonary disease
DVT: Deep vein thrombosis
PE: Pulmonary embolus
RAM: Risk assessment model
SCD: Sequential compression device
VTE: Venous thromboembolism
